# The extracellular lactate-to-pyruvate ratio modulates the sensitivity to oxidative stress-induced apoptosis via the cytosolic NADH/NAD^+^ redox state

**DOI:** 10.1007/s10495-020-01648-8

**Published:** 2020-11-23

**Authors:** Simei Go, Thorquil T. Kramer, Arthur J. Verhoeven, Ronald P. J. Oude Elferink, Jung-Chin Chang

**Affiliations:** 1 Tytgat Institute for Liver and Intestinal Research, Amsterdam UMC, University of Amsterdam, Amsterdam, The Netherlands; 2 Amsterdam Gastroenterology and Metabolism (AG&M) Research Institute, Amsterdam UMC, University of Amsterdam, Amsterdam, The Netherlands

**Keywords:** Warburg effect, Lactate, Pyruvate, Cytosolic NADH/NAD^+^ redox state, Raptinal, JNK

## Abstract

The advantages of the Warburg effect on tumor growth and progression are well recognized. However, the relevance of the Warburg effect for the inherent resistance to apoptosis of cancer cells has received much less attention. Here, we show here that the Warburg effect modulates the extracellular lactate-to-pyruvate ratio, which profoundly regulates the sensitivity towards apoptosis induced by oxidative stress in several cell lines. To induce oxidative stress, we used the rapid apoptosis inducer Raptinal. We observed that medium conditioned by HepG2 cells has a high lactate-to-pyruvate ratio and confers resistance to Raptinal-induced apoptosis. In addition, imposing a high extracellular lactate-to-pyruvate ratio in media reduces the cytosolic NADH/NAD^+^ redox state and protects against Raptinal-induced apoptosis. Conversely, a low extracellular lactate-to-pyruvate ratio oxidizes the cytosolic NADH/NAD^+^ redox state and sensitizes HepG2 cells to oxidative stress-induced apoptosis. Mechanistically, a high extracellular lactate-to-pyruvate ratio decreases the activation of JNK and Bax under oxidative stress, thereby inhibiting the intrinsic apoptotic pathway. Our observations demonstrate that the Warburg effect of cancer cells generates an anti-apoptotic extracellular environment by elevating the extracellular lactate-to-pyruvate ratio which desensitizes cancer cells towards apoptotic insults. Consequently, our study suggests that the Warburg effect can be targeted to reverse the lactate-to-pyruvate ratios in the tumor microenvironment and thereby re-sensitize cancer cells to oxidative stress-inducing therapies.

**Electronic supplementary material:**

The online version of this article (10.1007/s10495-020-01648-8) contains supplementary material, which is available to authorized users.

## Introduction

The enhancement of aerobic glycolysis (Warburg effect), first described by Otto Warburg in 1924 [1], supports tumorigenesis in numerous ways. First believed to be caused by dysfunctional mitochondria, it is now established to be the consequence of oncogene activation or tumor suppressor inactivation [2, 3]. While aerobic glycolysis maintains sufficient glycolytic intermediates for multiple biomolecule synthetic pathways [4], most of the carbon skeleton from glucose is secreted in the form of lactate and does not contribute to biomolecule synthesis [5]. In addition, increased flux through the pentose phosphate pathway provides the bulk of NADPH needed for lipid synthesis and neutralization of reactive oxygen species (ROS) [6]. The latter is important because many cancers display elevated levels of ROS and are in a state of constitutive oxidative stress that would result in apoptosis if not counterbalanced by NADPH-dependent defenses [7, 8]. On the other hand, the elevated ROS levels continuously drives DNA mutagenesis for tumor progression and stabilizes HIF-1α for high glycolytic capacity and angiogenesis [9–12]. Moreover, constitutive ROS-mediated signaling via the MAPK/ERK1/2 or PI3K/Akt/mTOR pathways promotes cell proliferation and survival [13, 14].

Resistance to apoptosis is one of the hallmarks of cancer [15]. Despite the usage of cytotoxic agents to specifically induce cancer apoptosis, therapeutic efficacy is counteracted by drug resistance, genomic instability and tumor heterogeneity [16]. Hence, combining cytotoxic agents with therapies targeting other essential biological processes, such as tumor growth, are preferred [17]. The recently characterized compound Raptinal induces apoptosis via oxidative stress in a wide variety of (cancer) cell lines in an unusually rapid fashion and attenuates tumor growth in in vivo tumor xenografts [18]. On the other hand, targeting of tumor metabolism by compounds such as dichloroacetate (DCA) and 3-bromopyruvate are attractive because of the various anti-tumor properties that accompany the inhibition or reversal of the Warburg effect [19, 20].

In the present study, we show that the Warburg effect generates an extracellular environment with an elevated lactate-to-pyruvate ratio, which protects against oxidative stress-induced apoptosis by reducing the cytosolic NADH/NAD^+^ redox state. Mechanistically, a reduced cytosolic NADH/NAD^+^ redox state inhibits oxidative stress-induced apoptosis by suppressing mitochondrial outer membrane permeabilization (MOMP) mediated by JNK-Bax signaling. Conversely, oxidizing the cytosolic NADH/NAD^+^ redox state sensitizes cancer cells to oxidative stress-induced apoptosis by enhancing JNK-Bax signaling.

## Materials and methods

### Cell lines and culture conditions

The hepatoma cell line HepG2 and colorectal carcinoma cell line HCT116 were maintained in DMEM (Invitrogen, Landsmeer) with 2 g/L glucose, 1.8 g/L NaHCO_3_, 20 mM HEPES–NaOH (pH 7.4), 10% fetal bovine serum (FBS), 100 U/mL penicillin and 100 µg/mL streptomycin under 5% CO_2_. The human immortalized cholangiocyte cell line H69 was cultured as described previously [21]. The leukemia cell lines U937 and HL-60 were maintained in IMDM (Invitrogen, Landsmeer) with 10% FBS, 100 U/mL penicillin and 100 µg/mL streptomycin under 5% CO_2_. For the generation of conditioned medium, 6.5 × 10^5^ cells were plated in 2 mL culture medium on 6-wells plates. Medium was subsequently harvested at 24, 48 and 72 h after seeding, filtered through 0.22 µm pore filter and stored at -20 °C until further use. All media used for experiments were pre-equilibrated overnight at 37 °C in the 5% CO_2_ incubator.

### Reagents

Base DMEM (no glucose, glutamine, phenol red, bicarbonate), digitonin, rotenone, Na-dichloroacetate, Na-l-lactate, Na-pyruvate, Na-chenodeoxycholate (Na-CDC), *N*-Acetyl-l-cysteine (NAC) and JNK inhibitor SP600125 were purchased from Sigma. Raptinal was purchased from BioVision. TNF-related apoptosis-inducing ligand (TRAIL) was purchased from R&D. Dihydroethidium (DHE) was purchased from Abcam. Antibodies against phosphorylated JNK (Thr183/Tyr185), JNK, Bax, cleaved caspase-3 and vinculin were obtained from Cell Signaling (Bioké, Amsterdam); anti-phosphorylated Bax (Thr167) was obtained from Assay Biotech (Tebu-Bio, Heerhugowaard), anti-PARP from Sigma, and anti-Tom20 and anti-cytochrome *c* from Santa Cruz (Bio-Connect, Huissen).

### Cell treatment for induction of apoptosis

Cells were cultured in 96-well plates until confluence and refreshed the day before the experiment. On the day of an experiment, cells were refreshed with 100 µL experimental medium or (un)conditioned culture medium. For suspension cells, 5 × 10^5^ cells per well were directly seeded in a 96-wells plate in experimental medium. The experimental medium consisted of base DMEM, supplemented with 5.5 mM glucose, 1.5 g/L NaHCO_3_, 20 mM HEPES–NaOH (pH 7.4), 10 µg/mL phenol red and 1% FBS. Cells were pre-incubated for 1 h in media containing different lactate-to-pyruvate ratios in the presence or absence of various pharmacological inhibitors. Following pre-incubation, cells were treated with vehicle (0.1% DMSO), 10 µM Raptinal, 200–750 µM Na-chenodeoxycholate or 40 ng/mL TRAIL in the respective medium for the indicated time points.

### Caspase 3/7 activity assay

Measurement of caspase 3/7 activity was performed as described in detail [21]. Briefly, by the end of incubation, combined activity of caspase 3 and caspase 7 was measured with the SensoLyte Homogeneous Rh110 Caspase 3/7 Assay Kit (Tebu-Bio, Heerhugowaard).

### Enzymatic determination of glucose, lactate and pyruvate

Spent medium was deproteinized by collecting 50 µL of spent medium in 75 µL of 5% metaphosphoric acid. Samples were incubated for at least 1 h on ice and subsequently spun down at 10,000*×g* for 10 min. Supernatant was collected and stored at − 20 °C until analysis. Medium glucose, lactate and pyruvate were enzymatically determined as described in [22] using the CLARIOstar microplate reader.

### Cloning of tetracycline inducible Peredox-mCherry vector and lentiviral transduction

Cloning of the Peredox-mCherry NADH/NAD^+^ redox sensor and lentiviral transduction of the sensor into cells was performed as described in [23].

### Clamping the cytosolic NADH/NAD^+^ redox state by lactate-to-pyruvate clamping solution

Clamping of the cytosolic NADH/NAD^+^ redox state, or [NADH]/[NAD^+^] ratio, was achieved by incubating cells in medium containing a fixed total amount (2.5 mM) of l-lactate plus pyruvate in different lactate-to-pyruvate ratios. Stock solutions of 100 mM Na-l-lactate and 100 mM Na-pyruvate were prepared in 50 mM NaCl.

### Measurement of cytosolic NADH/NAD^+^ redox state with Peredox-mCherry biosensor

HepG2 cells stably transduced with the inducible Peredox-mCherry construct or an empty vector (pCW-MCS-BSD) as described in [23]. Cells were induced with 800 ng/mL doxycycline for 48 h prior to the experiment. Cells were incubated in HBSS modified for ambient air medium containing a fixed total amount (2.5 mM) of l-lactate plus pyruvate but in different lactate-to-pyruvate ratios (1, 5, 10, 20, 50 and 100) in the presence of glucose. Fluorescence of Peredox (F_1_) and mCherry (F_2_) were monitored as described in [23]. Fluorescence from HepG2 cells transduced with the empty vector was used to correct for background fluorescence (denoted as F_1_′ and F_2_′). The background-corrected fluorescence ratio R was defined as (F_1_−F_1_′)/(F_2_−F_2_′). R was then linearly transformed by normalizing the minimal fluorescence ratio R_min_ (2.5 mM pyruvate in the absence of glucose) and the maximal fluorescence ratio R_max_ (2.5 mM l-lactate in the absence of glucose) to 100% and 200%, respectively.

### Superoxide anion radicals measurement by DHE oxidation

HepG2 cells were pre-incubated in phenol-red free DMEM containing 5.5 mM glucose and a fixed total amount (2.5 mM) of l-lactate plus pyruvate in different lactate-to-pyruvate ratios (1, 250) for 45 min at 37 °C. Subsequently, 5 µM DHE was added and after a 15-min incubation, 10 µM Raptinal (or vehicle, 0.1% DMSO) was added. Oxidation of DHE was monitored fluorometrically at λ_Ex_/λ_Em_ = 520 ± 10/590 ± 20 in the CLARIOstar microplate reader under 5% CO_2_ atmosphere. The slope of the changes in fluorescence between 30 and 60 min after Raptinal addition was used to calculate the oxidation rate of DHE.

### Cytochrome *c* release assay

Cytochrome *c* release assay was performed as previously described in detail [21]. Briefly, following incubation of the cells the plasma membranes were selectively permeabilized by incubation with 75 µg/mL digitonin dissolved in intracellular buffer at 4 °C for 20 min. Solubilized cytosolic proteins were harvested and the remaining fraction, containing mitochondria, were lysed in RIPA buffer. Equal volumes were loaded from each fraction for immunoblotting.

### Immunoblotting

Immunoblotting was performed as previously described in detail [21]. Transferred proteins on PVDF membranes were blocked in 5% milk overnight and incubated with primary antibody solutions. Following incubation with primary antibody, membranes were incubated with goat-anti-mouse or goat-anti-rabbit antibodies conjugated to horse-radish peroxidase. Finally, membranes were developed with a homemade enhanced chemiluminescence solution (100 mM Tris–HCl pH 8.5, 1.25 mM luminol, 0.2 mM p-coumarin and freshly added 3 mM H_2_O_2_) and chemiluminescence was recorded using the LAS4000 machine (GE healthcare, Eindhoven).

### Statistical analysis

All data are expressed as mean ± SD. Statistical significance was tested using the one or two-way ANOVA, followed by Sidak or Tukey multiple comparison tests using Graphpad Prism 8.0 software. p-values ≤ 0.05 were considered as significantly different.

## Results

### HepG2-conditioned medium protects against Raptinal-induced apoptosis

The human hepatoma cell line HepG2 is an established liver cancer cell model for apoptosis and exhibits a strong Warburg effect. The latter property is characteristic of fast-growing cancers and is associated with increased resistance to cytotoxic agents [19]. Therefore, we used HepG2 to study the influence of the cellular metabolic state on oxidative stress-induced apoptosis.

**Fig. 1:**
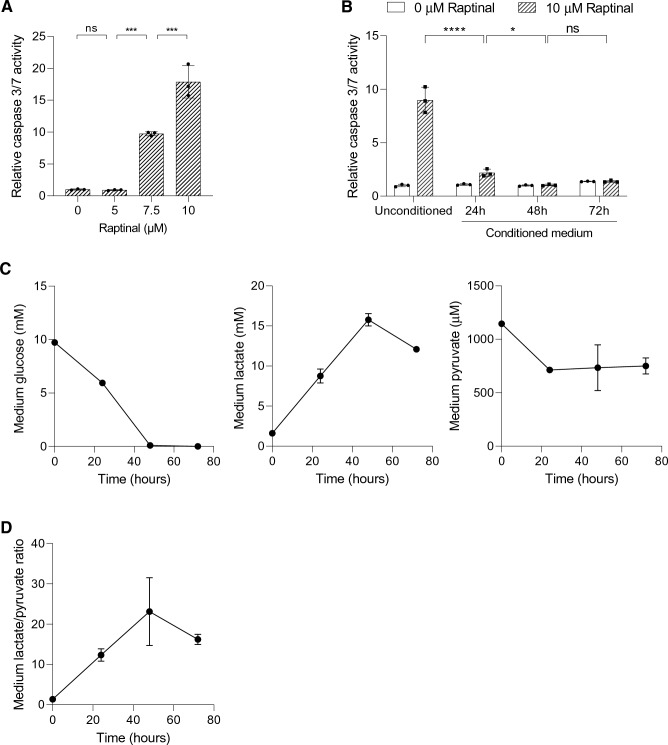
HepG2-conditioned medium protects against Raptinal-induced apoptosis. **a** HepG2 cells were treated with different concentrations of Raptinal for 1.5 h and caspase 3/7 activity was measured. Data is normalized to 0 µM Raptinal (vehicle, 0.1% DMSO) condition. Shown are the mean ± SD of a representative experiment (N = 2). One-way ANOVA with multiple comparison (Tukey) with ***p < 0.001, ns; not significant. **b** HepG2 cells were pre-incubated with the conditioned media for 1 h and thereafter treated with vehicle (0.1% DMSO) or 10 µM Raptinal for 1.5 h. Caspase 3/7 activity was then measured. Data is normalized to vehicle in unconditioned medium and shown are the mean ± SD of a representative experiment (N = 2). Two-way ANOVA with multiple comparison (Tukey) with ****p < 0.001, *p < 0.05, ns; not significant. **c** The medium glucose, lactate, and pyruvate of cultured HepG2 cells were measured at 24, 48 and 72 h after seeding. Shown are the mean ± SD of a representative experiment (N = 2). **d** The lactate-to-pyruvate (L/P) ratio in the medium conditioned by HepG2 cells over time. The ratios are derived from (**c**)

Raptinal is a rapid inducer of the intrinsic apoptotic pathway in a wide range of cancer cells [18]. Incubating HepG2 cells with Raptinal in fresh medium dose-dependently induced apoptosis as measured by caspase 3/7 activity (Fig. 1a). In contrast, incubation with Raptinal in conditioned medium from a parallel HepG2 culture dramatically suppressed the apoptotic response (Fig. 1b). These data strongly suggest that HepG2 cells can condition their extracellular environment to protect against oxidative stress-induced apoptosis.

We hypothesized that the conditioned medium contains or lacks factors that modulate the sensitivity to Raptinal-induced apoptosis. One prominent difference between conditioned and unconditioned medium is the accumulation of glycolytic end-products as a result of the Warburg metabolic phenotype of HepG2 cells. To confirm this phenotype in our model system, we measured the concentrations of glucose, pyruvate and lactate in the medium in HepG2 cultures over time (Fig. 1c). During culture, HepG2 cells converted the majority of glucose in the culture medium into lactate but reduced medium pyruvate, thereby elevating the medium lactate-to-pyruvate ratio over time (Fig. 1d). These findings demonstrated a correlation between the extracellular lactate-to-pyruvate ratio and the sensitivity to apoptosis.

### The extracellular lactate-to-pyruvate ratio controls the cytosolic NADH/NAD^+^ redox state

**Fig. 2:**
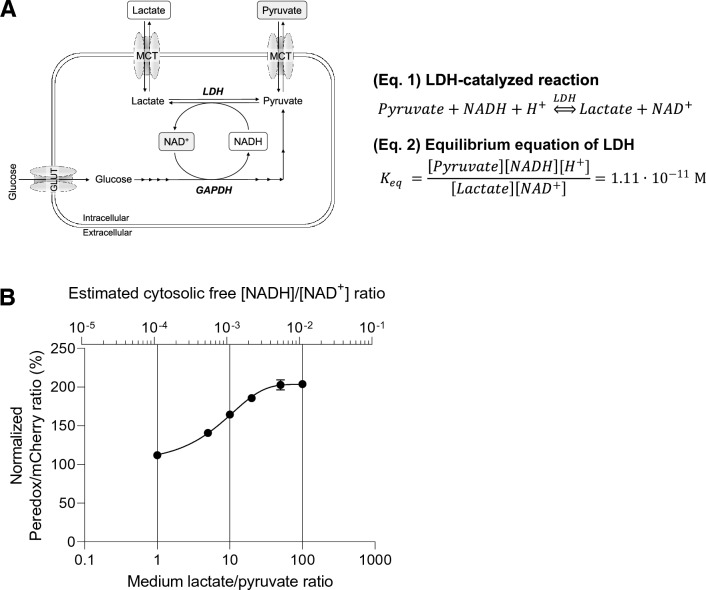
The extracellular lactate-to-pyruvate ratio controls the cytosolic NADH/NAD^+^ redox state. **a** The theoretical and schematic model of how extracellular L/P ratios affect intracellular cytosolic NADH/NAD^+^ redox state. Monocarboxylate transporters (MCTs) equilibrate extracellular lactate ([Lac]_ex_) and pyruvate ([Pyr]_ex_) with cytosolic lactate ([Lac]_c_) and pyruvate ([Pyr]_c_). Via the action of lactate dehydrogenase (LDH), the [Lac]_c_/[Pyr]_c_ is in near equilibrium with the cytosolic [NADH]_c_/[NAD^+^]_c_ ratio. Hence, [Lac]_ex_/[Pyr]_ex_ ≈ [Lac]_c_/[Pyr]_c_ ≈ [NADH]_c_/[NAD^+^]_c_. **b** Fluorescence ratio (R) of the cytosolic NADH/NAD^+^ Peredox biosensor to tandem-tagged mCherry in transduced HepG2 was monitored by incubating the cells with different extracellular ratios of lactate to pyruvate in the presence of glucose. The total amount of lactate and pyruvate was maintained at 2.5 mM. Data are normalized as described in Materials and Methods. Cytosolic free [NADH]/[NAD^+^] ratios are calculated using Eq. 2 as stated in (**a**) with K_eq_ derived from [24] and using [H^+^] of pH 7.0. Shown are the mean ± SD of a representative experiment (N = 2)

The extracellular lactate-to-pyruvate ratio is an established indicator of the cytosolic NADH/NAD^+^ redox state [24]. Via the monocarboxylate transporters (MCTs), extracellular lactate and pyruvate are in near equilibrium with the cytosolic pool of lactate and pyruvate. In turn, the cytosolic pool of lactate and pyruvate is in near equilibrium with the cytosolic free NADH and NAD^+^ via the reversible catalytic action of lactate dehydrogenase (LDH) (Fig. 2a) [24, 25]. Due to these metabolic interactions it is expected that the cytosolic free [NADH]/[NAD^+^] ratio can be clamped by manipulating the extracellular lactate-to-pyruvate ratio.

To verify that the extracellular lactate-to-pyruvate ratio directly alters the cytosolic free [NADH]/[NAD^+^] ratio, we expressed the [NADH]/[NAD^+^] redox biosensor Peredox in the cytosol in HepG2 cells. Incubations of HepG2 cells in media with a fixed total concentration (2.5 mM) of lactate and pyruvate, but at varying ratios ranging from low (oxidized cytosolic NADH/NAD^+^ redox state) to high (reduced cytosolic NADH/NAD^+^ redox state), resulted in a stable ratio-dependent change of Peredox fluorescence intensity over time (Figs. 2b; S1). These data show that the extracellular lactate-to-pyruvate ratio indeed controls the cytosolic free [NADH]/[NAD^+^] ratio. Assuming a cytosolic pH of 7.0 in HepG2 cells and the equilibrium constant of LDH, the cytosolic [NADH]/[NAD^+^] ratio can even be estimated from the extracellular lactate-to-pyruvate ratio (Fig. 2b).

### The extracellular lactate-to-pyruvate ratio modulates the sensitivity to Raptinal-induced apoptosis by regulating the cytosolic NADH/NAD^+^ redox state

**Fig. 3:**
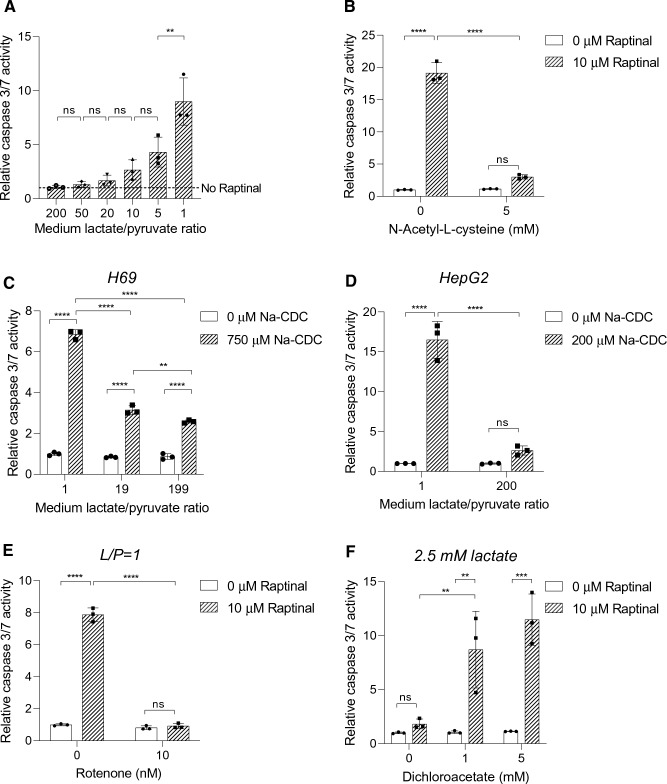
The cytosolic NADH/NAD^+^ redox state alters sensitivity to oxidative stress-induced apoptosis. **a** HepG2 cells were pre-incubated for 1 h in fresh media with different L/P ratios. Following pre-incubation, cells were treated with vehicle (0.1% DMSO) or 10 µM Raptinal for 1.5 h. Caspase 3/7 activity was determined and normalized to no Raptinal condition. Data represent mean ± SD of a representative experiment (N = 3). **b** HepG2 cells were pre-incubated with 5 mM NAC under an oxidizing cytosolic NADH/NAD^+^ redox state (L/P = 1) for 1 h. Then, vehicle (0.1% DMSO) or 10 µM Raptinal was added for 1.5 h and caspase 3/7 activity was measured. Data are normalized to vehicle condition and shown are the mean ± SD of a representative experiment (N = 3). **c** Caspase 3/7 activity assay of H69 cells pre-incubated for 1 h in media with different L/P ratios and then treated with vehicle (0.1% DMSO) or 750 µM Na-CDC for 1 h. Data are normalized to L/P = 1 vehicle condition and are representative of N = 3 experiments. **d** Similar treatment as in (**c**), only in HepG2 cells and with 200 µM Na-CDC. Data are normalized to L/P = 1 vehicle condition and are representative of N = 3 experiments. **e** HepG2 cells were pre-incubated for 1 h in the presence or absence of 10 nM rotenone, an inhibitor of complex I of the electron transport chain, under an oxidizing redox clamp (L/P = 1). Subsequently, cells were treated with vehicle (0.1% DMSO) or 10 µM Raptinal for 1.5 h and caspase 3/7 activity was measured at the end of incubation. Data are normalized to vehicle in the absence of rotenone. Shown are the mean ± SD from a representative experiment (N = 2). **f** HepG2 cells were pre-incubated with 0, 1, and 5 mM DCA for 1 h under reducing redox clamp (2.5 mM lactate). Subsequently, cells were incubated with vehicle (0.1% DMSO) or 10 µM Raptinal for 1.5 h and caspase 3/7 activity was measured at the end of incubation. Data are normalized to vehicle in the absence of DCA. Difference in salt concentration was compensated by NaCl. Total added salt concentration was fixed at 5 mM across incubations. Shown are mean ± SD from a representative experiment (N = 3). Statistical analysis: **a** one-way ANOVA with multiple comparison (Tukey) (**b**–**f**) Two-way ANOVA with multiple comparison (Tukey) with ****p < 0.0001, ***p < 0.001, **p < 0.01, *ns* not significant

Since HepG2-conditioned medium had an elevated medium lactate-to-pyruvate ratio and suppressed Raptinal-induced apoptosis (Fig. 1b, d), we next investigated whether the increased lactate-to-pyruvate ratio by itself confers protection against Raptinal-induced apoptosis. To this end, we incubated HepG2 cells with Raptinal in fresh culture media containing different lactate-to-pyruvate ratios ranging from 1 (oxidized cytosolic NADH/NAD^+^ redox state) to 200 (reduced cytosolic NADH/NAD^+^ redox state) in the presence of glucose. Indeed, elevating the lactate-to-pyruvate ratio in fresh medium stepwise desensitized HepG2 cells to Raptinal-induced apoptosis (Fig. 3a). Since the cytosolic free [NADH]/[NAD^+^] ratio is directly affected by the medium lactate-to-pyruvate ratio (Fig. 2b), these results strongly suggest that a high cytosolic free [NADH]/[NAD^+^] ratio, i.e. a more reduced cytosolic NADH/NAD^+^ redox state, confers resistance to Raptinal-induced apoptosis.

It has been reported that Raptinal induces apoptosis by imposing oxidative stress [18]. Therefore, we investigated the effect of anti-oxidant *N*-acetyl-l-cysteine (NAC) on Raptinal-induced apoptosis. Indeed, NAC effectively suppressed Raptinal-induced apoptosis (Fig. 3b), confirming that Raptinal induces apoptosis via oxidative stress. To exclude that the protective effect of the cytosolic NADH/NAD^+^ redox state is exclusive for Raptinal we used the hydrophobic bile salt sodium chenodeoxycholate (Na-CDC) as another inducer for oxidative stress-induced apoptosis [26]. We therefore treated H69 cholangiocytes, previously shown to be sensitive to bile salt-induced apoptosis [21], and HepG2 cells with Na-CDC in medium containing different lactate-to-pyruvate ratios. Elevation of the extracellular lactate-to-pyruvate ratio proved to be protective against bile salt-induced apoptosis both in H69 cholangiocytes (Fig. 3c) and in HepG2 cells (Fig. 3d). These data strongly imply that the cytosolic NADH/NAD^+^ redox state not only modulates the sensitivity to Raptinal-induced apoptosis, but affects oxidative stress-induced apoptosis in general.

We next examined whether Raptinal-induced apoptosis is affected by manipulations of the cytosolic [NADH]/[NAD^+^] redox other than by clamping the extracellular lactate-to-pyruvate ratio. Cytosolic NADH is primarily generated by glycolysis and oxidized in the cytosol to NAD^+^ by LDH, or shuttled into mitochondria via malate-aspartate or glycerol-3-phosphate shuttles. Since mitochondria are the main consumers of NADH, sustained manipulation of mitochondrial function (in)directly influences the cytosolic free [NADH]/[NAD^+^] ratio. Indeed, it has been reported that lactate secretion and the cellular [NADH]/[NAD^+^] redox ratio are elevated upon inhibition of complex I of the mitochondrial respiratory chain [27]. In contrast, promotion of mitochondrial pyruvate oxidation by dichloroacetate, a pyruvate dehydrogenase kinase inhibitor, has been shown to decrease the cellular [NADH]/[NAD^+^] redox ratio [28]. We used these conditions and tested if rotenone can protect against apoptosis by increasing the cytosolic free [NADH]/[NAD^+^] ratio. We indeed observed a protection against apoptosis by rotenone (Fig. 3e). Conversely, we tested if decreasing the cytosolic [NADH]/[NAD^+^] ratio by co-incubation with DCA re-sensitized cells to apoptosis. Indeed, DCA re-sensitized HepG2 cells to Raptinal-induced apoptosis even in the presence of a high lactate-to-pyruvate ratio (Fig. 3f). Together, these results support the notion that the cytosolic NADH/NAD^+^ redox state regulates oxidative stress-induced apoptosis.

### The extracellular lactate-to-pyruvate ratio regulates Raptinal-induced apoptosis by inhibition of JNK activation

**Fig. 4:**
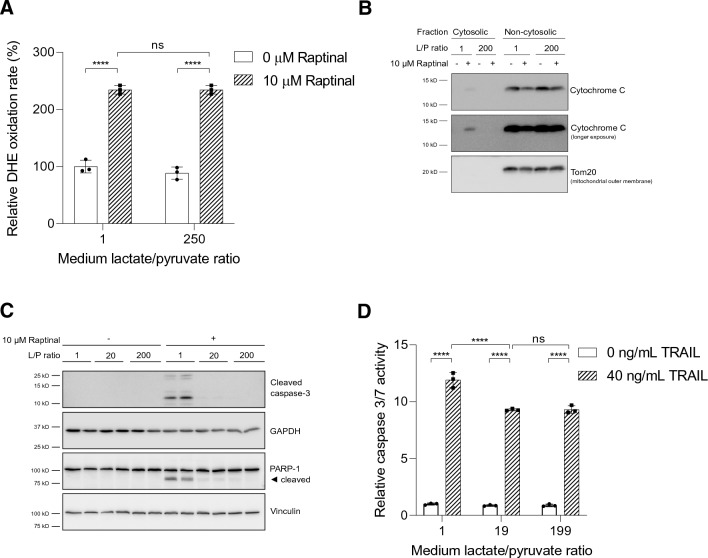
A reduced cytosolic NADH/NAD^+^ redox state suppresses intrinsic apoptosis induced by oxidative stress. **a** HepG2 cells were incubated in medium with L/P ratios of 1 and 250 for 1 h prior to addition of Raptinal (or vehicle, 0.1% DMSO). The oxidation rate of DHE was monitored fluorometrically. Rates of DHE oxidation were normalized to vehicle-treated cells under oxidized redox clamp (L/P = 1) set to 100%. Data are represented as mean ± SD and are representative of two independent experiments. **b** HepG2 cells were pre-incubated for 1 h in media containing different L/P ratios. Following pre-incubation, vehicle (0.1% DMSO) or 10 µM Raptinal was added for 2 h. Cytosolic fractions were separated from non-cytosolic fractions (containing mitochondria). To assess cytochrome *c* release from mitochondria, equal volumes of cytosolic and non-cytosolic fractions were immunoblotted for cytochrome *c*. Shown is a representative experiment of N = 3. **c** Treatment as in (**b**) but whole cell lysates were prepared and immunoblotted for cleaved caspase-3 and its substrate, PARP-1. Shown is a representative experiment of 3 independent experiments (N = 3). **d** HepG2 cells were pre-incubated for 1 h in media with different L/P ratios after which 40 ng/mL TRAIL was added for 4 h. Caspase 3/7 activity was then measured. Data are normalized to vehicle condition under L/P = 1 and presented as mean ± SD of a representative experiment (N = 3). Statistical analysis (**a**, **d**): two-way ANOVA with multiple comparison (Tukey) with ****p < 0.0001, *ns* not significant

We and others have shown that Raptinal induces apoptosis by imposing oxidative stress (Fig. 3b) [18]. We therefore investigated whether the extracellular lactate-to-pyruvate ratio affects Raptinal-induced production of ROS. To this end, we measured the generation of superoxide anion radicals under oxidized and reduced cytosolic NADH/NAD^+^ redox state. We observed no effect of the medium lactate-to-pyruvate ratio on the generation of superoxide anion radicals by Raptinal (Fig. 4a). These data indicated that a high extracellular lactate-to pyruvate ratio, and therefore a reduced cytosolic NADH/NAD^+^ redox state, acts downstream of ROS production to protect against Raptinal-induced apoptosis.

As Raptinal has been reported to induce apoptosis through the intrinsic mitochondrial pathway, we examined whether the cytosolic NADH/NAD^+^ redox state regulates cytochrome *c* release, which is required to initiate the caspase activation cascade in intrinsic apoptosis. Indeed, Raptinal induced release of cytochrome *c* into the cytosol in cells clamped under an oxidized cytosolic NADH/NAD^+^ redox state, but not under a reduced cytosolic NADH/NAD^+^ redox state (Fig. 4b). Next, we investigated the effect of different cytosolic NADH/NAD^+^ redox states on cleavage of caspase-3 and the caspase-3 substrate PARP-1. Consistently, we observed the cleavage of caspase-3 and PARP-1 under an oxidized cytosolic NADH/NAD^+^ redox state by Raptinal, but not under a reduced cytosolic NADH/NAD^+^ redox state (Fig. 4c). Interestingly, clamping cells under a reduced cytosolic NADH/NAD^+^ redox state did not protect cells from apoptosis by TNF-related apoptosis-inducing ligand (TRAIL), an activator of the extrinsic pathway of apoptosis (Fig. 4d) [29]. These results indicate that the cytosolic NADH/NAD^+^ redox state only regulates the intrinsic mitochondrial apoptotic pathway.

**Fig. 5:**
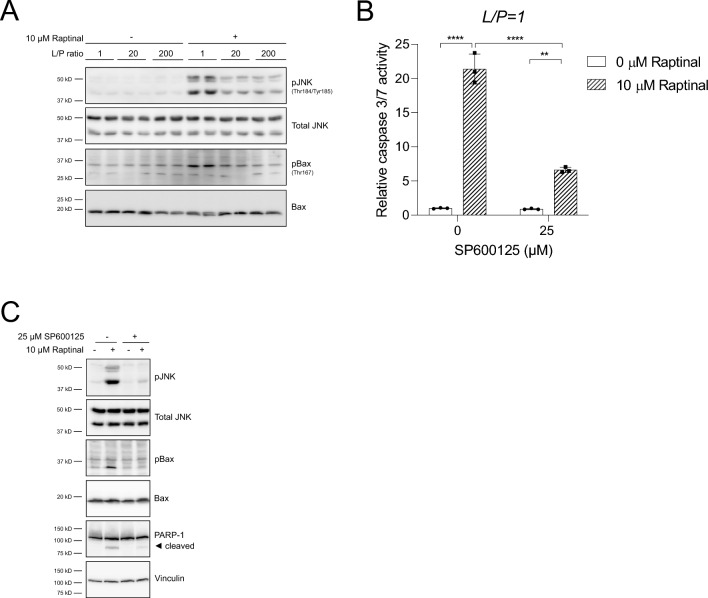
A reduced cytosolic NADH/NAD^+^ redox state suppresses Raptinal-induced apoptosis by inhibiting JNK activation. **a** HepG2 cells were pre-incubated for 1 h and clamped in media with different L/P ratios after which vehicle (0.1% DMSO) or 10 µM Raptinal was added for 2 h. Whole lysates were prepared and immunoblotted for p-JNK, and p-Bax. Shown is a representative experiment of N = 3. **b** HepG2 cells were pre-incubated for 1 h with the JNK inhibitor SP600125 under oxidizing redox clamp (L/P = 1). Subsequently, vehicle (0.1% DMSO) or 10 µM Raptinal was added for 1.5 h. At the end of the incubation, caspase 3/7 was measured. Data are normalized to vehicle in absence of SP600125 and shown are the mean ± SD of a representative experiment of N = 2. Statistical significance was tested with a two-way ANOVA with multiple comparison (Tukey) with ****p < 0.0001, **p < 0.01. **c** Treatment as in (**b**) but for 2 h with vehicle (0.1% DMSO) or 10 µM Raptinal in the presence and absence of SP600125. At the end of incubation, cells were harvested and immunoblotted for p-JNK, p-Bax, and PARP-1. Shown is a representative experiment of N = 2

Whilst we have verified that Raptinal induces apoptosis via the intrinsic mitochondrial pathway by generating oxidative stress (Figs. 3b, 4a) [18], the mechanism has not been characterized in detail. JNK is known to promote different forms of oxidative stress-induced apoptosis by coordinating the activation of Bax, an effector protein for MOMP, and several BH3-only Bcl-2 proteins, such as Bim, and Bmf [30, 31]. Therefore, we examined whether Raptinal induces activation of JNK and used the phosphorylation of Bax at Thr167 as a readout for JNK-dependent activation of MOMP proteins [32]. Indeed, we observed a clear increase of JNK and Bax phosphorylation by Raptinal under an oxidized cytosolic NADH/NAD^+^ redox state, which was strongly decreased under a reduced cytosolic NADH/NAD^+^ redox state (Fig. 5a). To test if JNK activation is an obligatory step for Raptinal-induced apoptosis, we treated HepG2 cells with the JNK inhibitor SP600125 and found that JNK inhibition strongly decreased Raptinal-induced apoptosis under an oxidized cytosolic NADH/NAD^+^ redox state (Fig. 5b). Furthermore, JNK inhibition not only effectively prevented phosphorylation of Bax at Thr167, but also prevented activation of JNK itself in HepG2 cells (Fig. 5c). As observed in HepG2 cells, similar protective effects of a reduced cytosolic NADH/NAD^+^ redox state and of the JNK inhibitor SP600125 were noted on Raptinal-induced apoptosis in U937 and HL-60 cells (Fig. S2A, B). In contrast, the sensitivity of HCT116 cells to Raptinal-induced apoptosis was hardly affected by reducing the cytosolic NADH/NAD^+^ redox state or inhibition of JNK (Fig. S2C). Taken together, our results demonstrate that JNK mediates Raptinal-induced apoptosis and that the cytosolic NADH/NAD^+^ redox state regulates oxidative stress-induced apoptosis downstream of ROS production, but upstream of the activation of JNK.

## Discussion

Accumulation of lactic acid in the tumor microenvironment is typical of tumors that exhibit a strong Warburg effect. Many aerobic glycolytic cancers have increased expression of *LDHA*, which preferentially converts pyruvate into lactate and thereby increases lactate secretion but decreases pyruvate secretion [33]. We demonstrate here that this high lactate but low pyruvate extracellular environment elevates the cytosolic free [NADH]/[NAD^+^] ratio (Fig. 2b), which is in line with the observation that cancerous tissues have an elevated cytosolic free [NADH]/[NAD^+^] ratio in comparison to healthy (adjacent) tissue [34, 35]. We further show that this elevated cytosolic NADH/NAD^+^ redox state confers resistance to oxidative stress induced apoptosis by Raptinal via decreased JNK activation (Fig. 5a).

**Fig. 6:**
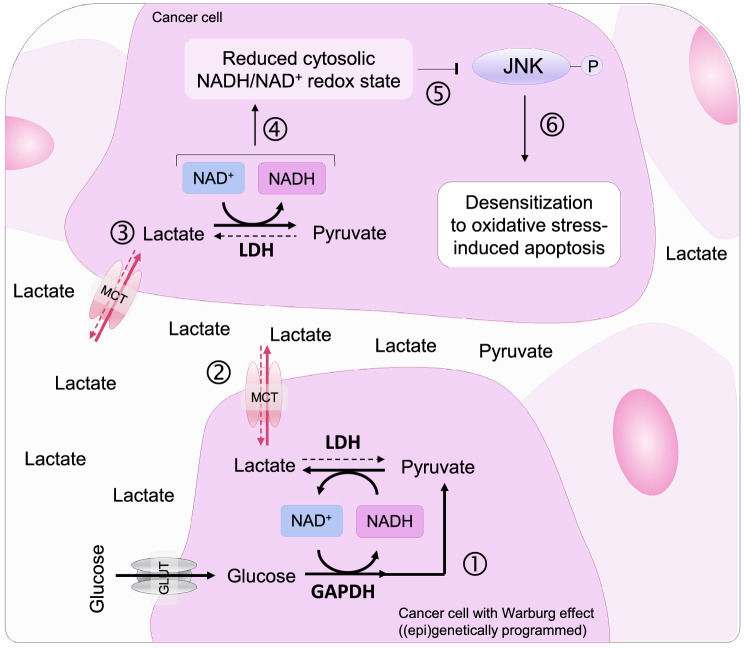
The Warburg effect desensitizes cancer cells from oxidative stress-induced apoptosis. The Warburg effect of cancer cells is characterized by increased glucose uptake, glycolysis and lactate formation (1). Intracellular lactate is transported out of the cells by MCTs, which increases the lactate-to-pyruvate ratio (L/P) in the extracellular environment (2). Both lactate and pyruvate enter neighboring cancer cells via MCTs and regulate the cytosolic NADH/NAD^+^ redox state through LDH (3). Due to the excess extracellular lactate over pyruvate (i.e. high extracellular L/P ratio), the cytosolic NADH/NAD^+^ redox state of cells is clamped in a reduced state (i.e. high cytosolic [NADH]/[NAD^+^] ratio) (4). This reduced cytosolic NADH/NAD^+^ redox state inhibits JNK activation under oxidative stress (5) and desensitizes cancer cells to oxidative stress-induced apoptosis (6)

Extracellular lactate levels in the internal tumor core can increase by approximately 20–40 times in comparison to healthy tissues [36, 37]. Increased lactate content in cancer tissue correlates with resistance to radiotherapy in solid tumors [38]. Moreover, administration of lactate has been shown to be protective in different cellular models of apoptosis [39, 40] but the metabolic background of this phenomenon has not been studied. It has been reported that cholangiocarcinoma and colorectal cancer cells maintain low pyruvate by levels by oncogenic c-myc-enhanced expression of *LDHA* and *PKM2* and by limiting pyruvate entry through epigenetic silencing of *SLC5A8*. This decrease in intracellular pyruvate activates HDAC1/3-mediated transcriptional upregulation of anti-apoptotic proteins Bcl2 and survivin, and downregulation of pro-apoptotic proteins p53 and Bax [41–43]. However, it is unlikely that transcriptional regulation is responsible for the protective effect observed in our study because of the rapid action of Raptinal (within 2 h). In vivo, it is possible that prolonged increased lactate and decreased pyruvate exposure (high lactate-to-pyruvate ratio) and subsequent changes on the cytosolic NADH/NAD^+^ redox state and transcriptional regulation synergistically protect cancers from apoptosis. Moreover, via “paracrine” signaling, lactate may also affect the cytosolic NADH/NAD^+^ redox state and confer resistance to oxidative stress-induced apoptosis in cells that are not (epi)genetically programmed to exhibit the Warburg effect (Fig. 6).

Our data indicate signaling via the oxidative stress sensor JNK as an important effector for modulating the sensitivity of Raptinal-induced apoptosis exerted by the cytosolic NADH/NAD^+^ redox state (Fig. 5a). Under oxidative stress, JNK-mediated phosphorylation of 14–3-3, a cytoplasmic anchor of Bax, and downstream Bcl2-family proteins including Bax itself, leads to liberation and mitochondrial translocation of Bax to initiate MOMP [30, 32, 44]. In HCT116 cells, Raptinal is capable of inducing cytochrome *c* release even in the absence of the essential components (Bak/Bax/Box) required for canonical pore formation [45], suggesting a yet unidentified mechanism for Raptinal induced cytochrome *c* release in these cells. Consistently, our data indicate that this unidentified mechanism is rather insensitive to changes in the cytosolic [NADH]/[NAD^+^] ratio or JNK inhibition (Fig. S2C). Changes of the cytosolic [NADH]/[NAD^+^] ratio also had no effect on TRAIL-induced cleavage of Bid (data not shown), which is then capable of bypassing the JNK-dependent Bax regulation and initiate MOMP [29]. The lack of a protective effect of a reduced cytosolic NADH/NAD^+^ redox state (and JNK inhibition) in HCT116 cells by Raptinal or in HepG2 under TRAIL-induced apoptosis imply that canonical JNK-Bax signaling of the intrinsic mitochondrial pathway is pivotal for its protective effect. Interestingly, we also observed decreased Raptinal-induced JNK phosphorylation in HepG2 cells treated with the JNK inhibitor SP600125, which is consistent with the reported positive feedback loop of JNK signaling under oxidative stress [46, 47].

Apart from the decrease in JNK activation by Raptinal under a Warburg effect environment, we did not directly identify the target(s) responsible for the protective effect. However, because of its rapid action, the protective effect likely involve a post-translational regulation that is sensitive to manipulations of the cytosolic NADH/NAD^+^ redox state. Recently, Sarikhani et al. demonstrated that deacetylation of JNK by cytosolic NAD^+^-dependent SIRT2 activates the kinase activity of JNK and thereby promotes oxidative stress-induced apoptosis [48]. This is consistent with the protective effect of SIRT2 inhibition described in other models of oxidative stress-induced apoptosis [49–51]. However, since cytosolic free [NAD^+^] by far exceeds [NADH] (700:1), a small change in [NAD^+^] would have large effect on [NADH]. This makes NADH a far more sensitive indicator of the cytosolic [NADH]/[NAD^+^] redox ratio than NAD^+^. The buildup of NADH in cancers is proposed to competitively inhibit NADPH-dependent thioredoxin-reductase, which is important to sustain the phosphatase action of tumor suppressor Phosphatase and tensin homolog (PTEN). Inactivation of PTEN leads to Akt activation and resistance to arsenic trioxide-induced apoptosis [19, 52]. Indeed, lactate-induced Akt activation protected cancer cells from glucose starvation-induced apoptosis [39]. However, supplementation of lactic acid still protected colon cancer cells from pan-Akt inhibitor uprosertib-induced apoptosis, suggesting that lactate-mediated apoptosis protection may also occur independent of Akt signaling [53]. Future studies are required to further characterize the effects of the cytosolic NADH/NAD^+^ redox state, potentially exerted via SIRT2 or PTEN, on JNK and its modulatory effect on apoptosis.

Finally, pharmacological inhibitors of mitochondria such as rotenone and DCA, which (indirectly) affect cytosolic NADH/NAD^+^ redox state in opposite directions, reversed the sensitizing and desensitizing effects of oxidized and reduced cytosolic NADH/NAD^+^ redox state, respectively (Fig. 3e, f). Consistently, high-throughput compound screening with the NADH/NAD^+^ biosensor SoNar showed that compounds lowering cytosolic free [NADH]/[NAD^+^] ratio are associated with cancer cell cytotoxicity [35]. In addition, restoration of the pyruvate transporter *SLC5A8* in colorectal cancer cells sensitizes them to pyruvate-induced apoptosis whereas pyruvate administration restored the cytotoxic effects of doxorubicin, 5-fluorouracil, and oxaliplatin in otherwise chemo-resistant cancer cells.

In summary, we show that the crosstalk between metabolism and apoptosis can be exploited to overcome resistance to anti-cancer therapy, where the balance of extracellular metabolites (pyruvate and lactate) acts as a determinant of apoptosis tolerance. This apoptosis tolerance, which is dependent on the cytosolic NADH/NAD^+^ redox state and the activation of key enzyme JNK under oxidative stress, may also be relevant for other stimuli that induce oxidative stress, including chemotherapeutic agents. Consequently, oxidizing the cytosolic NADH/NAD^+^ redox state by pyruvate supplementation or by pharmacological inhibitors such as DCA may aid as adjuvant therapy in sensitizing cancer cells with an intact apoptotic signaling machinery to oxidative stress-inducing therapies.

## Electronic supplementary material

Below is the link to the electronic supplementary material.

Electronic supplementary material 1 (DOCX 13 kb)

Electronic supplementary material 2 (PDF 110 kb)

## Data Availability

Raw data is available on request.
