# Correction: The extracellular lactate-to-pyruvate ratio modulates the sensitivity to oxidative stress-induced apoptosis via the cytosolic NADH/NAD + redox state

**DOI:** 10.1007/s10495-024-02024-6

**Published:** 2024-10-30

**Authors:** Simei Go, Thorquil T. Kramer, Arthur J. Verhoeven, Ronald P. J. Oude Elferink, Jung-Chin Chang

**Affiliations:** 1 Tytgat Institute for Liver and Intestinal Research, Amsterdam UMC, University of Amsterdam, Amsterdam, The Netherlands; 2 Amsterdam Gastroenterology and Metabolism (AG&M) Research Institute, Amsterdam UMC, University of Amsterdam, Amsterdam, The Netherlands

**Correction to: Apoptosis (2021) 26:38–51**

10.1007/s10495-020-01648-8

In Fig. 5C of the article, the labels (10 µM Raptinal, 25 µM SP600125) of the immunoblot were accidentally swapped during preparation of the manuscript. The figures should have appeared as shown below. The interpretation of the results and the accompanying text and figure legends in the original manuscript were correct and have not been adapted.

**Fig. 5:**
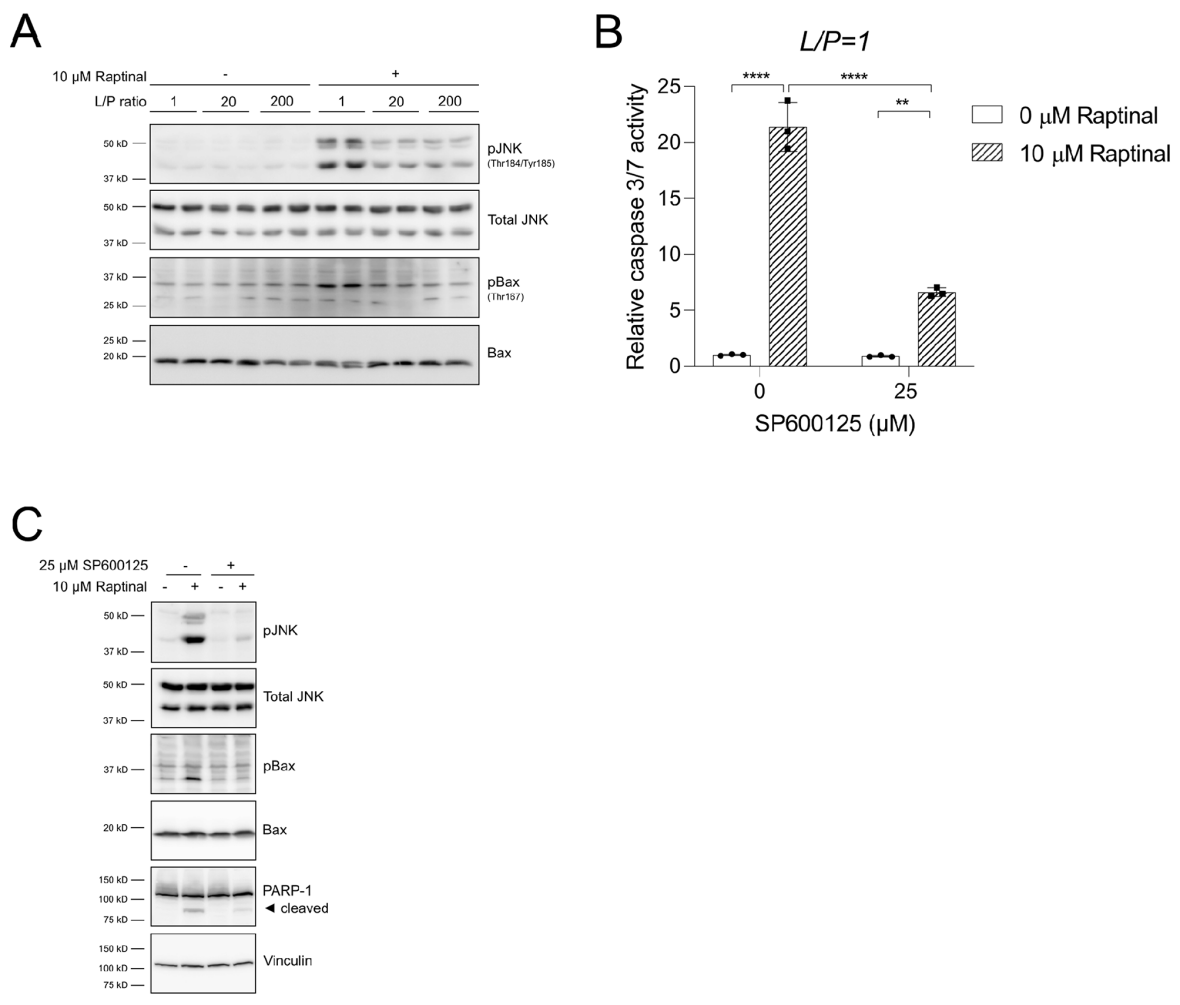
A reduced cytosolic NADH/NAD^+^ redox state suppresses Raptinal-induced apoptosis by inhibiting JNK activation. **a** HepG2 cells were pre-incubated for 1 h and clamped in media with different L/P ratios after which vehicle (0.1% DMSO) or 10 μM Raptinal was added for 2 h. Whole lysates were prepared and immunoblotted for p-JNK, and p-Bax. Shown is a representative experiment of N = 3. **b** HepG2 cells were pre-incubated for 1 h with the JNK inhibitor SP600125 under oxidizing redox clamp (L/P = 1). Subsequently, vehicle (0.1% DMSO) or 10 μM Raptinal was added for 1.5 h. At the end of the incubation, caspase 3/7 was measured. Data are normalized to vehicle in absence of SP600125 and shown are the mean ± SD of a representative experiment of N = 2. Statistical significance was tested with a two-way ANOVA with multiple comparison (Tukey) with ****p < 0.0001, **p < 0.01. c Treatment as in (**b**) but for 2 h with vehicle (0.1% DMSO) or 10 μM Raptinal in the presence and absence of SP600125. At the end of incubation, cells were harvested and immunoblotted for p-JNK, p-Bax, and PARP-1. Shown is a representative experiment of N = 2

The Original article has been corrected.

