# Supplementary material for: The extracellular lactate-to-pyruvate ratio modulates the sensitivity to oxidative stress-induced apoptosis via the cytosolic NADH/NAD+ redox state
Source: Apoptosis. 2020 Nov 23;26(1-2):38–51. doi: 10.1007/s10495-020-01648-8 (PMC7902596; doi:10.1007/s10495-020-01648-8)
Supplement: Supplementary file 1 — Electronic supplementary material 1 (DOCX 13 kb) [file 10495_2020_1648_MOESM1_ESM.docx]

**Legends for Supplemental Figures**

**Supplemental Figure 1**

HepG2 cells were incubated in media with different L/P ratios prior to measurement. Next, fluorescence of Peredox-mCherry was monitored over time as described in Methods. Shown here are the background-corrected, non-normalized Peredox-mCherry ratios. Data are representative of two independent experiments. The data points in the shaded gray area were used to generate Fig. 2B.

**Supplemental Figure 2**

Raptinal treatment of U937 (A), HL-60 (B), and HCT116 (C) cells under an oxidized (L/P=1) or reduced (L/P=200) cytosolic [NADH]/[NAD^+^] ratio, or under an oxidized clamp (L/P=1) in the presence of JNK inhibitor SP600125. Data are normalized to the L/P=1 vehicle condition. Statistical analysis (A-C) Two-way ANOVA with multiple comparison (Tukey) with **** p<0.0001, ** p<0.01, *p<0.05, ns; not significant.
