# Supplementary figures and images for: The extracellular lactate-to-pyruvate ratio modulates the sensitivity to oxidative stress-induced apoptosis via the cytosolic NADH/NAD+ redox state
Source: Apoptosis. 2020 Nov 23;26(1-2):38–51. doi: 10.1007/s10495-020-01648-8 (PMC7902596; doi:10.1007/s10495-020-01648-8)

# Supplemental Figure 1

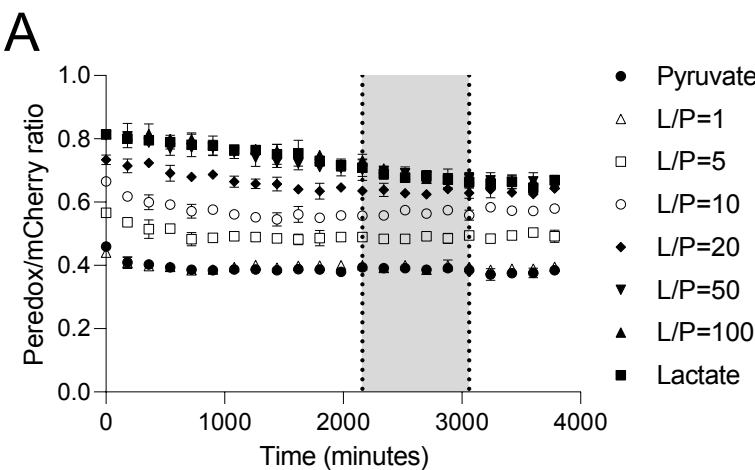

# Supplemental Figure 2

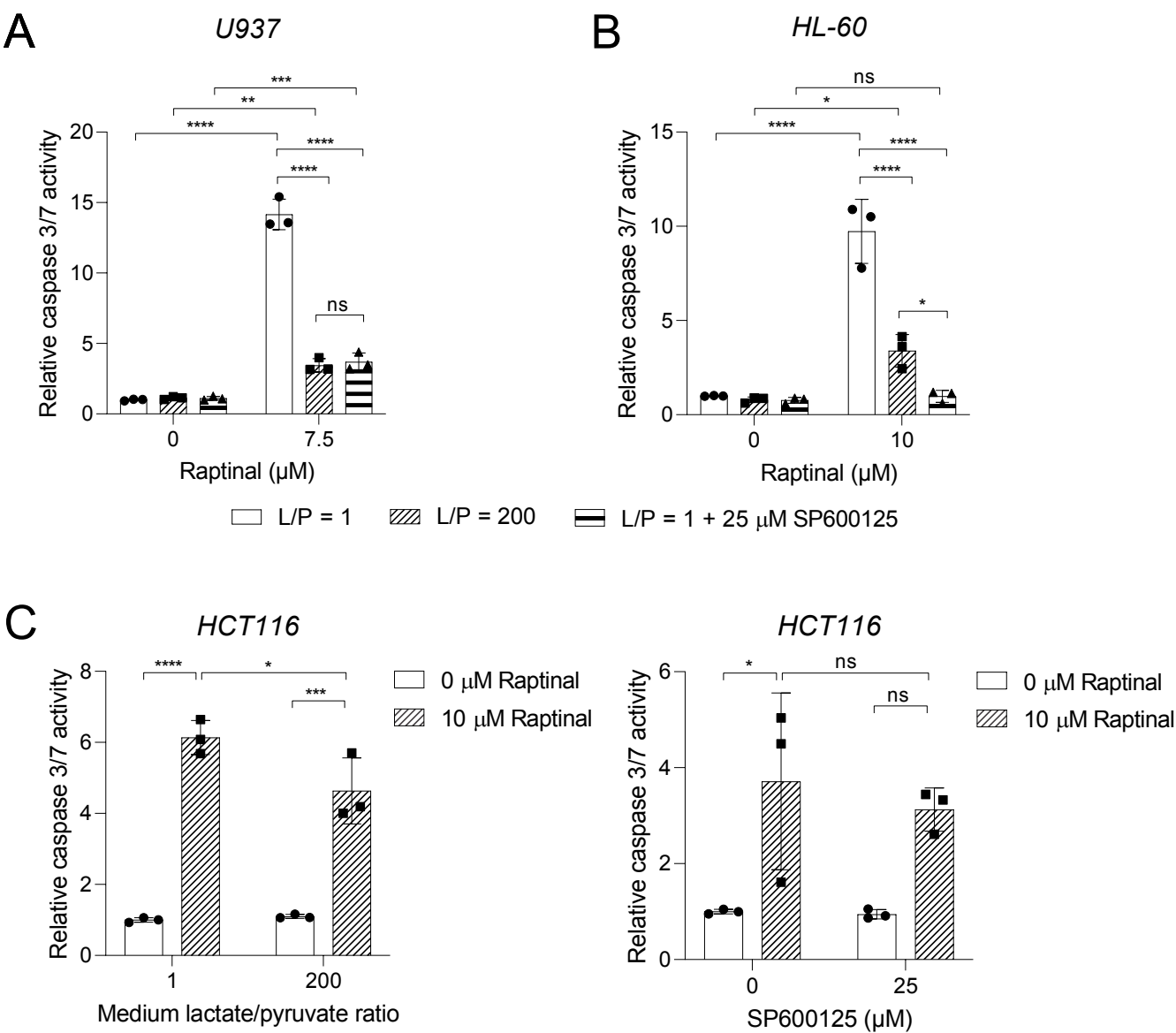

Supplement: Supplementary file 2 — Electronic supplementary material 2 (PDF 110 kb) [file 10495_2020_1648_MOESM2_ESM.pdf]
